# A Distributed Effects Perspective of Dimensionally Defined Psychiatric Disorders: And Convergent Versus Core Deficit Effects in ADHD

**DOI:** 10.3389/fpsyt.2014.00062

**Published:** 2014-06-05

**Authors:** T. Sigi Hale

**Affiliations:** ^1^Department of Psychiatry and Biobehavioral Sciences, UCLA Semel Institute for Neuroscience and Human Behavior, Los Angeles, CA, USA

**Keywords:** ADHD, attention, brain-state, hemispheres, laterality, model, network, theory

## Abstract

The focus of psychiatric and psychological research has arguably shifted from brain damage and psychosis to more common forms of psychopathology that reflect extremes variants of otherwise normal cognitive and behavioral characteristics. Now, in addition to trying to understand overtly damaged brain-function (flat tire effects), we are also seeking to understand liabilities associated with non-optimized, but otherwise intact, cognitive and behavioral abilities (poor tuning effects). This shift has pushed us to evolve our investigational strategies to more broadly consider whole-brain integrated brain systems, as well as seek to develop more specific quantifiable indicators of impoverished brain function and behavior. This paper discusses such challenges in relation to dimensionally defined psychiatric disorders and presents a novel whole-brain integrated perspective of ADHD brain function pathology.

## Preface

This work begins with an examination of a set of highly theoretical concepts (see New Age in Psychiatric Research, Three Levels of Interest: Modular, Functional, and Brain-State Setting, and A Multi-Level Deficit Analysis Schema in Section Setting the Stage – An Exploration of Ideas), and then applies those concepts to the construction of a more formal and rigorous model presentation of ADHD brain function pathology (see The TD-APS Brain System, TD-APS Distributed Effects and ADHD, and Model Predictions and Future Studies in Section The Model). This first section falls outside the boundaries of what is typically expected in scientific works, as it is essentially a contemplating of ideas. Nevertheless, we felt it necessary to do this in order to generate the needed conceptual framework for our model presentation. We hope the reader will indulge this initial highly theoretical exploration, and consider it a type of background to the subsequent and more formally developed work.

## Setting the Stage – An Exploration of Ideas

### A new age in psychiatric research

**Figure 1:**
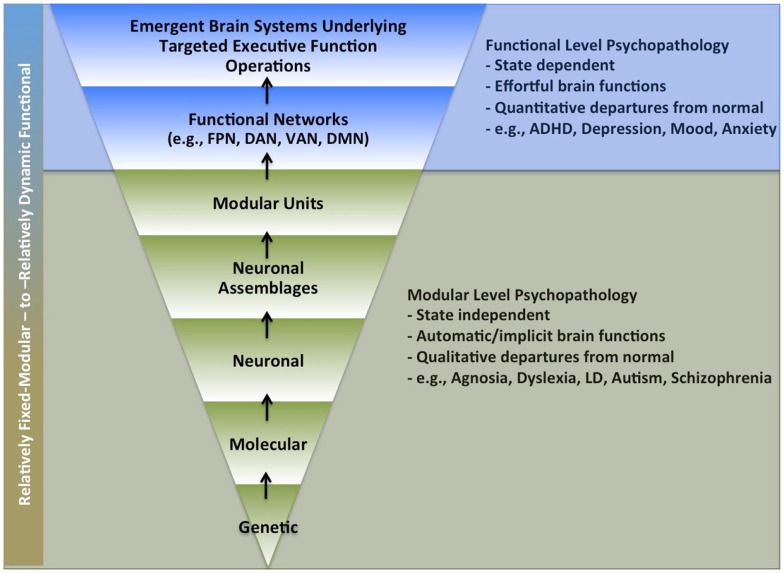
**Possible levels of impairment underlying psychiatric disorders**. *Legend*: multiple levels of brain function levels that might underlie brain function pathology in psychiatric disorders. Lower section (in green) highlights levels more likely to involve automatic processing (i.e., that do not require self-directed effort). Upper section (in blue) highlights levels more likely to require active volitional effort. FPN, fronto-parietal network; DAN, dorsal attention network; VAN, ventral attention network; DMN, default mode network; LD, learning disability.

Human brain function involves multiple integrated scales of operations, with each passing its computational outputs to subsequent and higher levels. This means that as one’s investigational focus moves up and out toward manifest thought, experience, and behavior, the scope and complexity of underlying brain mechanisms necessarily increases as it must include all constituent lower-level operations. This circumstance is particularly relevant to psychiatric research. Like psychology, this field contends with disordered brain function at the upper bounds of complex human thought and behavior, but unlike psychology, it also seeks to elucidate and treat associated neurobiological mechanisms. This presents the extremely daunting challenge of having to consider possible neurobiological deficits across all constituent layers and scales of human brain functioning (i.e., from genes to behavior) (Figure 1).

Moreover, whereas psychiatric research once largely focused on psychopathology that involved overt qualitative departures from normal behavior and experience (e.g., brain-injury and psychoses), it has now come to emphasize the study of more dimensionally defined disorders that reflect extreme variants of otherwise normal cognitive and behavioral characteristics (e.g., ADHD, depression, anxiety, mood dysregulation, etc.). This shift has even further complicated the challenges inherent to psychiatric research. Now, in addition to trying to understand mechanisms underlying overtly damaged brain function (“flat tire effects”), we are also seeking to understand liabilities associated with non-optimized, but otherwise intact, cognitive, and behavioral abilities (“poor tuning effects”).

Progress in this domain will likely require the continued development of investigational schemas that allow us to better conceptualize interactive impairment effects across multiple levels and scales of brain functioning. Mesulam (1) was an early proponent of this goal. He highlighted three distinct but highly integrated levels of brain function (modular, distributed, and state setting), and characterized the difficulties inherent to distinguishing causal versus secondary cascading effects across these levels. He also implied that the relative modularity of brain operations could, to some extent, be inferred from their anatomical scale (i.e., local versus distributed networks). We have found distinguishing fixed-modular and functional-emergent effects in this way to be conceptually difficult, as all biological operations possess both characteristics, albeit at different anatomical scales and time courses. Accordingly, we have attempted to devise and will now present our own provisional approach to this conceptual challenge, as well as the challenge of considering interactive deficit effects across multiple levels and scales of brain function operations.

### Three levels of interest: Modular, functional, and brain-state setting

Human brain function is understood to involve both modular “building block” type operations, as well as more functional-emergent aspects whereby modular units get flexibly integrated to serve ongoing cognitive and behavioral challenges. Although one cannot draw absolute conceptual boundaries around these different classes of brain function, we have found it useful to emphasize their relative automaticity to make such distinctions. This is based on the contention that cognitive effort is likely tied to the induction and maintenance of more functional-emergent aspects. Hence, brain functions requiring effort are considered more functional, while those that do not are considered more fixed and modular. Under this view, even distributed network effects can, in theory, reflect fixed-modular processing to the extent they produce automatic computational outputs, while local networks could, in theory, produce functional-emergent effects to the extent their operations depend on applied cognitive effort. Nevertheless, as Mesulam suggests, we acknowledge there is typically a positive relationship between the anatomical scale of distributed networks and the prevalence of functional-emergent effects.

Mesulam’s work also highlighted the importance of brain-state setting mechanisms. Specifically, he suggested both local and distributed network elements are subjected to modulation via chemically addressed channels that adjust arousal states and provide a type of syntax or matrix within which processing occurs. In this vein, we think it an intriguing possibility that such brain-state setting operations might include a capacity to flexibly reorganize (i.e., tune) the brain’s internal computational structures to serve unique classes of cognitive and behavioral actions, and this concept is gaining support (2–4).

To clarify, imagine a highly advanced automobile that is able to automatically re-tune its underlying mechanical systems so they become optimized for different driving conditions (e.g., leisure driving, snow driving, off-roading, high-speed cornering, drag racing, etc.). It would be like having multiple cars in one. Moreover, while the cars fixed mechanical substrates would remain constant across such shifts in tuning, the more functional-emergent aspects would be expected to change. For example, the car’s suspension should be tuned quite differently for leisure driving versus high-speed cornering. Lastly, such states should also constrain the nature and extent of the driver’s top-down influence. For example, the driver should have more finessed control over the car’s performance under a high-speed cornering versus a leisure driving mode.

We think human volitional brain-state setting operations may reflect a similar dynamic whereby brain functions, potentially at all levels and scales, get functionally re-configured to serve distinct genres of cognitive and behavioral actions (e.g., task-directed, exploration, creativity, and emergency response, etc.). Under this view, the executor (i.e., the self) not only influences the unfolding of real-time neuro-computational events (i.e., top-down executive functions, EFs), but also what adaptive processing state (APS) (i.e., brain-state) gets brought to bear to face those challenges.

In summary, building upon Mesulam’s work, we have highlighted three levels of brain function operations: fixed-modular, functional-emergent networks, and brain-state setting. We have chosen to conceptually distinguish modular and functional aspects based on the degree to which their operations depend upon active volitional effort. Moreover, we have conceptualized that brain-state setting operations may include a capacity to dynamically “re-tune” the brain’s internal computational structures to suit different genres of cognitive and behavioral challenges, and consider this to be a unique expression of top-down influence. Specifically, we distinguish two levels. First, top-down influence bears on the induction and maintenance of an appropriate APS; then, within that brain-state, it bears on task and situation specific cognitive and behavioral actions (i.e., EFs). In the following section, we will describe our developing investigational schema for the assessment of psychiatric impairment across these described levels of brain function.

### Multi-level deficit analysis schema

This schema highlights the view that dimensionally defined psychiatric illnesses, such as ADHD, can result from deficits originating at any of the above discussed three levels of brain function, with associated unique consequences. It also highlights that primary deficits at any of these levels are expected to produce secondary cascading effects that transfer between them (Table 1). Below, we present possible UNIQUE characteristics of such deficits and “deficit flows” at each of the three described levels {[i.e., fixed-modular, functional-emergent, APS (brain-state) setting]}.

**Table 1:** **Multi-level deficit analysis schema for dimensionally defined disorders**.

	Deficit source considerations (three levels of pathology)	Manifest cognitive and behavioral capacity		
		Subclinical	Subclinical	Clinical
		***↑***	***↑***	*****↑*****
	**(3) APS effects**			
	Brain-state setting effects			*****↑*****
	Psychosocial + Cog. impairments	Compensated	Compensated	**Deficit**
	State-specific deficits			
Effortful-functional quantitative effects	**(2) Functional-emergent effects**			
	EF-level network effects		***↑***	
	Network-specific impairments	Compensated	**Deficit**	Normal
	State-dependent deficits			
Automatic qualitative	**(1) Fixed modular effects**			
	Mechanistic effects	***↑***		
	Mechanism-specific impairments	**Deficit**	Normal	Normal
	State-independent deficits			

*Shows three conceived levels of possible brain function pathology. Qualitative refer to deficits that involve overt departure from normal behavioral characteristics. Quantitative refers to deficits defined by “out of bounds” functioning of otherwise normal characteristics. Red path shows uncompensated “deficit flows” moving into higher-order brain functions and eventually manifesting clinical pathology, which necessarily impacts the upper most level (i.e., APS Effects). EF, executive function; APS, adaptive processing state; Cog., cognitive*.

#### Fixed-modular deficit effects

Fixed-modular deficit effects are conceptualized to reflect *broken* rather than *poorly tuned* brain functioning. Associated impairments are expected to be linked to specific lesion-like mechanical insults, and produce automatic brain-state independent deficits that reflect overt departures from normal abilities (i.e., flat tire effects). Moreover, because fixed-modular units are expected to serve as computational building blocks for multiple higher-order operations, uncompensated deficits at this level are expected to have diffuse secondary effects, potentially impacting multiple EF-level networks and/or APSs.

#### Functional-emergent deficit effects

Functional-emergent deficit effects are conceptualized to reflect integration difficulties *within* functional-emergent networks that support EF-level operations, for example: the dorsal and ventral attention, or fronto-parietal networks (DAN, VAN, and FPN) (5). Problems within such networks are expected to impact multiple EFs and/or APSs. They are considered brain-state *dependent* and *functional-emergent* to the extent their operations depend on volitional effort; however, such networks are also identified at rest, which suggests modular aspects. One possibility is that highly utilized EF-level network operations become increasingly automated over time to boost efficiency. If so, the relative intactness of such networks at rest may index a type of relative use history. Regardless, for this level of brain function analysis, we mean to only include functional-emergent aspects of EF-level network operations (i.e., that are associated with volitional effort), and conceptualize any associated modular components as belonging to the modular effects level.

#### Adaptive processing state (i.e., brain-state) deficit effects

Adaptive processing state deficit effects reflect impairment at the upper-bound of human brain function complexity where multiple EF-level networks and their associated modular operations get dynamically integrated to form emergent brain systems, which are optimized for key genres of cognitive and behavioral action (e.g., task-directed, exploration, creativity, emergency response, etc.). Problems at this level are expected to reflect difficulties with the *formation/maintenance* and/or *use* of such states. Here, “use effects” are meant to highlight that even with underlying brain functions intact, an individual might fail to “normally” adopt situation-appropriate APSs for primarily psychosocial reasons. Lastly, no matter the cause, abnormal functioning at this level is expected to be a necessary antecedent to the external manifestation of dimensionally defined psychiatric disorders. This is based on two lines of reasoning. First, APSs represent the highest possible level of human brain function operations that direct complex naturally occurring cognition and behavior (i.e., it is where the neuro-computational rubber meets the cognitive and behavioral road). Next, dimensionally or quantitatively defined psychiatric illnesses, such as ADHD, are specifically defined in relation to manifest complex psychosocial abilities linked to this upper echelon of brain function operations.

#### Deficit flows

Lastly, Table 1 also highlights the concept of deficit flow and the importance of distinguishing primary from secondary cascading effects (1). For example, a primary deficit at the fixed-modular level may cause secondary functional impairments at the higher levels, and if so, represents a more serious “biological brain problem” than one where the deficit originates directly at the APS level, even though both circumstances may produce similar clinical outcomes. Likewise, primary deficits at higher levels can create the appearance of lower-level impairments. For example, even though a modular brain function may be intact, it might not get properly integrated into some complex EF-level network operation and/or APS, creating the impression of a fixed-modular impairment, whereas it is actually a secondary functional-emergent effect.

Disentangling such possibilities is made difficult by several factors. For instance, deficit flows may or may not leave a “deficit trail”; that is, while uncompensated impairments should manifest detectable pathology at the level of deficit origination, sub-clinical weaknesses, and/or partly compensated deficits may not, and yet still contribute to, or even cause, manifest impairments at higher levels. Moreover, as noted, the complexity of brain-to-behavior pathways necessarily increases as you move into higher-order functions. Hence, abnormal brain function at the level of APS operations might reflect a dizzying array of underlying causal factors. With this conceptual framework, the only expected common feature of dimensionally defined psychiatric disorders is the poor functioning of a syndrome-defining APS and the general consequences from that, while the manner in which that brain system gets disrupted is expected to be variable and linked to syndrome heterogeneity and comorbidity. We believe ADHD reflects just such a circumstance.

#### The deficit analysis schema and ADHD

Unlike disorders such as agnosia or dyslexia, ADHD is linked to a particular form of complex psychosocial challenge; that is, a requirement to perform future-oriented complex task-directed actions under conditions of low intrinsic reward. Our basic supposition is that this ability depends on a specialized APS that facilitates complex task-directed actions (TD-APS), and that the syndrome-defining features of ADHD reflect the general consequences of this state’s associated brain functions being compromised, no matter the cause. We expect primary/causal deficits to the TD-APS can originate at any of the above described levels of brain function, with variability of *deficit origination and flow* underlying ADHD clinical heterogeneity and comorbidity.

**Figure 2:**
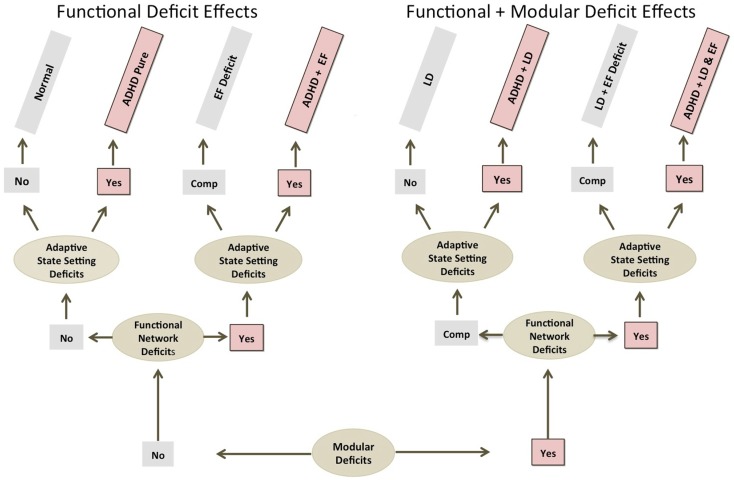
**ADHD comorbidity and primary deficit source considerations**. This shows how ADHD comorbidity profiles might help elucidate levels of brain function pathology underlying ADHD. Note: regardless of causal deficit sources, all ADHD producing etiological paths involve impairment at the adaptive state setting level. EF, executive function; LD, learning disorder; Comp., compensated.

Figure 2 below highlights how ADHD pathology might get initiated at different levels of brain function, and how this might be reflected in different comorbidity profiles. The basic premise is that primary brain function weakness at different levels should be associated with comorbidities reflective of brain function at that level. For example, modular level deficits should produce modular level comorbidities (e.g., learning disabilities); functional-emergent EF-level network deficits should produce EF-level comorbidities (e.g., anxiety and/or mood disorder), APS level deficits should produce pure ADHD, or rather, clinical symptoms exclusively tied to the improper functioning of the TD-APS (Figure 2).

In addition to consideration of comorbidities, we expect that analysis of deficit stability may also provide clues about the level of origination of impairments. Specifically, greater deficit stability may indicate more fixed-modular effects, while more variable and/or state-specific expression, may indicate more functional-emergent effects. Lastly, as noted, the nature of manifest clinical symptoms may be informative, with more fixed-modular impairments producing more qualitatively defined pathologies (i.e., flat tire effects), and more functional-emergent impairments producing more quantitatively defined pathologies (i.e., poor tuning effects).

In summary, our investigational schema is based on the view that normal human brain functioning requires top-down regulation of specialized brain-states that facilitate key genres of cognitive and behavioral actions (i.e., APSs), and that impairment of such states, no matter the cause, manifests clinical symptoms reflective of that state’s primary cognitive and behavioral objectives. Moreover, we have highlighted three key levels of brain functioning and presented some basic concepts regarding how deficits at each of those levels might be distinguished (i.e., comorbidity profiles, relative stability, and quantitative versus qualitative nature). Using this framework, we then indicated that disruption of a task- or goal-specialized APS (i.e., the TD-APS), no matter the cause, likely underlies common syndrome-defining ADHD clinical features, while variability in the source of TD-APS system impairment likely underlies ADHD clinical heterogeneity (including variably expressed comorbidities). We will now further consider what brain function operations are likely to comprise this proposed TD-APS state.

## The Model

### The TD-APS brain system

At a broad phenomenological level, being a skilled taskmaster requires an ability to internally organize the iterative steps of an intended plan-sequence and then mobilize sensory encoding operations in a manner that is tightly bound to the performance of that sequence. To this end, organizing, maintaining, and flexibly updating planned operations can be viewed as the TD-APS brain system’s primary *internal* computational goal, while identifying task-relevant sensory content with maximum efficiency can be viewed as its primary *external* computational goal. For this latter aspect, this means making accurate categorical discriminations of task-relevant sensory items using the minimum sensory exposure required to do so. Consciously engaging visual content beyond that boundary (i.e., task-irrelevant stimuli) reflects processing inefficiencies in relation to task-objectives, and is characterized here as “visual–sensory overflow.” All brain functions comprising the TD-APS brain system are conceived to be oriented toward these primary internal and external objectives.

#### The nodes

**Figure 3:**
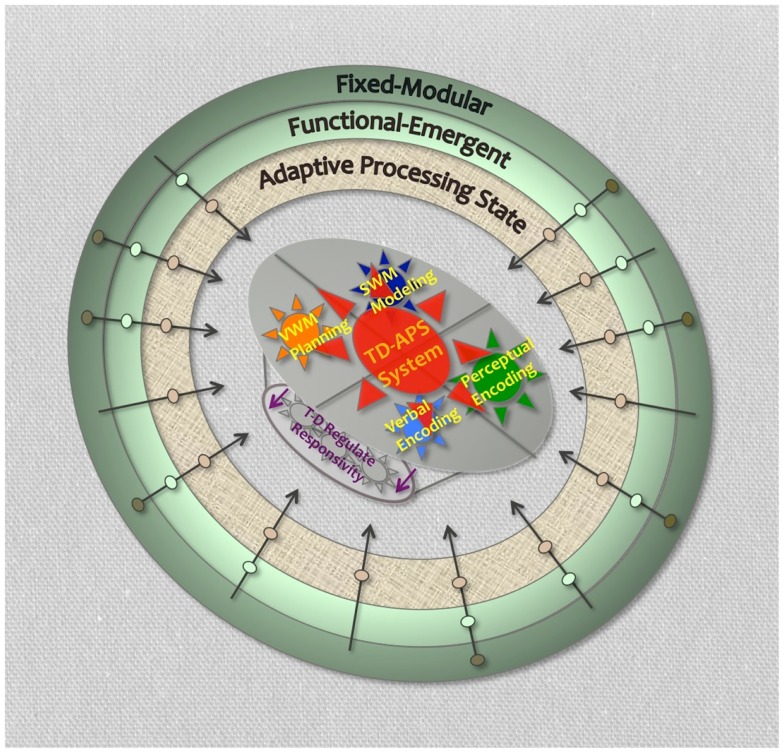
**Task-directed-adaptive processing state brain system**. Legend: shows four primary TD-APS brain system computational nodes. Outer rings represent levels of brain function that could impact TD-APS brain system. Arrows crossing outer rings represent hypothetical unique impairment trajectories with causal deficits originating at different levels of brain function. Purple arrows represent TD-APS intrinsic regulation of automatic sensory responsivity.

We conceive of four major computational nodes that are critical to the TD-APS brain system: (1) verbal working memory (VWM) for sequencing, maintaining, and updating task-directives (i.e., plans, instructions, rules, etc.), (2) visual–spatial working memory (SWM) for the internal modeling of sensory expectations to help bias down-stream processing toward task-objectives (also assists with node 1 operations-described below), (3) perceptual-level identification of task-relevant content, and (4) translation of this content into verbal codes that can be efficiently integrated with, and used to update, task-directives in VWM. This system covers four essential task-operations: planning, sensory modeling, perceptual encoding, and verbal encoding, and has the primary external objective of asserting top-down task-directed control over ongoing information processing and associated brain functions. Lastly, the successful induction and performance of this system is expected to require the active regulation of more automatic forms of sensory responsivity (Figure 3).

#### Connecting the nodes

There is nothing particularly new presented here about the proposed functionality of these nodal units. In fact, a great deal of work has already characterized how they interact to facilitate key forms of cognitive and behavioral operations (e.g., selective attention, predictive sensory/motoric modeling, visual search, behavioral inhibition, naming, etc.). What is new is the tying of them together, along with associated EF-level network operations, to form a coherent TD-APS brain system that can help us to: (1) contextualize variable ADHD cognitive impairments, (2) model ADHD clinical heterogeneity and comorbidity, given possibly diverse deficit sources within this proposed system, and (3) model common ADHD characteristics, given the proposed shared effects of poor system functioning, regardless of deficit sources.

#### Nodes 1 and 2 – the working memory functions

Desimone and Duncan (6) and Baddeley (7) were early proponents of the view that working memory (WM) was critical for operations beyond the traditionally understood role. For instance, Baddeley et al. (8) identified a central executive component that he suggested helps support selective attention operations, as well as an episodic buffer that might be involved with generating predictive internal imagery to guide task-operations. Even earlier, Desimone and Duncan (6) described WM as being related to the use of attention-templates that act as task-blueprints to help guide task-operations, namely the identification of task-relevant visual sensory content. These views have garnered much support. Recent studies show WM indeed plays dual roles, one that is internal analytic (i.e., the traditional concept of WM), as well as an applied role whereby it actively guides the selection of task-relevant content (9), and abundant research now links WM and selective attention abilities (10–13). This association between WM and selective attention underlies the proposed interactive dynamics of nodes 1 and 2 in our model, which we will now discuss.

Phonetic/articulatory codes seem an efficient means to dynamically represent, organize, and sequence multiple distinct pieces of information, and consistent with this, tasks that require such processing have consistently been shown to rely on left-lateralized VWM, for example: planning (14), top-down rule directed aspects of selective attention (13), rule based task switching (8, 15–18), and rule derived category learning (19). In contrast to this, right-lateralized frontal lobe functions appear to be associated with operations that should benefit more from perceptually represented content, such as: SWM (20), mental manipulation of objects (21), self-referential mental imagery (22), generating sensory and motoric predictive imagery to help guide ongoing actions (23), holistic integration of plan sequences (14), and integrative/perceptual aspects of category learning (19, 24).

Our conceptualization of these left/right differences is based on the following theoretical view. We conceive that the operative feature of the human two-hemisphere brain design may simply be that each hemisphere has become specialized to represent information using distinct data formats. Specifically, at the point at which sense data becomes explicitly represented, the left-hemisphere (LH) uses an abstract “verbal format” that links information to phonetic/articulatory codes, while the right hemisphere (RH) uses a “perceptual format” that attempts to generate a re-creation of sense data as it was originally perceived, or that is adjusted to a task-adaptive level of detail. We expect all brain functions utilize both formats in parallel; however, those better served by verbally indexed content are expected to show relative LH specialization, while those better served by perceptually coded content are expected to show relative RH specialization. Lastly, we point out that although very early implicit bottom-up processing of sense data occurs bilaterally, top-down influence at these early stages is expected to be right-biased for reasons that will be further discussed below.

According to this view, lateralized aspects of WM can be understood to reflect distinct “internal cognition work-spaces” that operate using different data formats (i.e., verbal and perceptual). For the purposes of our model, we suggest complex goal-directed actions utilize both work-spaces to help create, maintain, apply, and update plan sequences, albeit in different ways. The verbal work-space is thought to create and utilize a verbal index of intended task steps, while the perceptual work-space helps facilitate this operation by also dynamically modeling their integrative dynamics (i.e., when required and/or beneficial to do so) (14). Moreover, once an intended sequence is settled, the verbal cognition work-space is then conceived to manage and initiate iterative task steps, while the perceptual cognition work-space is thought to model associated predictive imagery or sensory expectations to help bias down-stream encoding toward task-objectives. These aspects of goal-directed brain function cover nodes 1 and 2 of our proposed TD-APS brain system. We will now further address how these operations are linked to the active perceptual identification of task-relevant content, and what EF-level networks are likely involved in that process.

#### Connecting nodes 1–2 to 3

In the course of performing task-directed actions, visual encoding must be constrained in two vital ways. First, exposure to details (encoding depth) must be constrained to facilitate fast categorical discriminations of task-relevant and irrelevant items, without undue exposure to unneeded sensory details (i.e., that are not strictly needed to make categorical judgments). Next, the scope of visual processing (encoding distribution) must be constrained so that it remains tightly bound to task-objectives. Mechanisms related to both functions are now discussed.

##### Encoding depth

During most task-operations, individuals are oriented toward quickly identifying task-relevant content and using that information in a specific task-prescribed manner. This represents a unique form of sensory encoding aimed at parsing sense data into categorical constructs so that determinations of task-relevance can be quickly made. It is fundamentally different than encoding operations utilized, for instance, to watch a sunset or enjoy a symphony, where the primary objective is to have a sensory *immersive experience*. Indeed, unless a task-operation directly calls for immersive experience, engagement of visual details beyond what is minimally required to make accurate categorical judgments is antithetical to most task-operations.

Hochstein and Ahissar (25) have presented an innovative model that suggests how appropriate visual encoding depth in relation to task-objectives might be achieved. This model details a “reverse hierarchy theory of visual processing” that addresses how visual encoding fluxes between states that facilitate the rapid parallel capturing of “the gist of visual scenes” with relative blindness to details (vision at a glance), versus the effortful linear scrutiny of visual details (vision with scrutiny). The model suggests pre-conscious encoding of visual information moves in a bottom-up manner through early processing stages, but that initial conscious perception occurs in higher areas that integrate multiple inputs to generate a cursory overview depiction of sensory items, which facilitates fast categorical parsing of sensory data (vision at a glance). Then, with effortful attention, lower-level visual areas can be further mined, via abundant re-entrant feedback connections, to extract additional details as needed (vision with scrutiny). Given that most task-operations benefit from the fast identification of task-relevant content, we expect “vision at a glance” mechanisms, as described, are likely critical and beneficial to the TD-APS brain system, while “vision with scrutiny” mechanism are more likely to be task-disruptive.

##### Encoding distribution

Hochstein’s model speaks to how a task-appropriate “encoding depth” might be achieved; however, there remains the matter of how individual sensory items get selected or suppressed in relation to task-objectives (i.e., encoding distribution). Bar (26) presented a model of visual encoding that speaks to this latter issue. Like Hochstein, they found early visual encoding involves an initial “low spatial frequency” representation, but further indicated that this representation gets projected to orbital prefrontal regions where an initial guess is made about its categorical identity, which is then back-projected onto temporal-occipital regions for further analysis (if needed). Bar’s model also identifies associated mechanisms involving parahippocampal, retrosplenial, and inferior frontal brain regions, which are suggested to play a role in tying the low frequency representations to “context frames” that bear knowledge from previous experience to help illuminate their identities [also see Ref. (27)]. In short, Bar’s model echoes Hochstein’s assertions regarding an initial “vision at a glance” type operation, but refers to these as “low frequency representations” and further delineates mechanisms associated with their fast automatic categorization.

It seems reasonable to expect that such automatic categorizations and associated assessments of item relevance must be regulated by top-down mechanisms during task-operations in order to remap “what is” and “is not” relevant based on specific task-objectives (i.e., not general biologic-behavioral relevance). Although the exact mechanisms underlying such top-down regulation are not yet elucidated, recent studies indicate that identified dorsal and ventral attention networks (VAN) likely play a role [for review see Ref. (2)].

The DAN involves bilateral frontal, parietal, and visual areas, and is thought to be associated with the top-down allocation of attention in space. The VAN is strongly right-lateralized, involving RH inferior frontal gyrus, anterior insula, and the temporal–parietal junction (TPJ), and is thought to be associated with the assessment of object relevance in relation to ongoing cognitive and behavioral operations (2), or more generally, with the testing and updating of internal processing states in relation to ongoing behavior (28). Moreover, both networks are understood to share a right-lateralized middle frontal gyrus component that may facilitate their interactive dynamics (2).

Multiple recent studies indicate that during task-operations the VAN must be regulated in a manner that allows the task-relevance of sensory items to take precedent over general biologic-behavioral relevance. Consistent with this, increased VAN activation, mainly in the RH TPJ, has been linked to off-task processing of sensory stimuli, and/or difficulty managing peripheral information (2, 5, 28–33). Moreover, multiple lines of research have shown that automatic perceptual encoding operations exhibit RH specialization, often overlapping with the VAN (namely the RH TPJ), such as: detection of sequence breaking novel objects (34), general assessments of object relevance (within our outside of task parameters) (35), and automatic perceptual/integrative category learning (19, 24). Together, these findings suggest that hyper-activation of right-lateralized VAN related systems, namely the RH TPJ, may index a weakened ability to assert task-directed top-down control over determinations of stimulus relevance.

Finally, multiple studies have indicated that RH frontal–parietal connectivity, likely involving DAN and VAN contributions, is critical for asserting top-down task-directed control over ongoing visual processing. Heilman et al. (36), Pardo and Raichle (37), and Corbetta et al. (38) presented early reports of RH specialized attention functions, and more recently, transcranial magnetic stimulation (TMS) studies have strongly linked RH frontal–parietal circuitry to selective attention for items in WM, and have shown EEG alpha plays a key role in facilitating RH frontal–parietal connectivity (10–12). Furthermore, recent work has linked sensory predictive modeling and selective attention, and highlighted that both operations critically rely on RH frontal–parietal circuitry [for review see Ref. (23, 39, 40)]. There is also evidence suggesting behavioral inhibition operations rely on this same circuitry [for review see Ref. (41, 42)].

Together, the above studies suggest RH biased perceptual encoding operations are critical for both automatic categorizations, and top-down selective processing of task-relevant stimuli. In relation to our proposed TD-APS brain system, this is consistent with the view that RH frontal brain regions support a type of perceptual cognition work-space where internal “low frequency” models of perceived stimuli can be matched against a library of previously experienced content to support automatic categorizations of sense data, and/or where task-specific sensory predictions/expectations can be volitionally modeled and used to bias down-stream processing toward task-objectives. Moreover, we expect that during task processing this system relies on a “vision at a glance” state orientation, integrates both dorsal and ventral attention networks, and is used across multiple behavioral domains (i.e., sustained attention, selective attention, modeling sensory expectations, and behavioral inhibition). Lastly, the above studies also suggest that difficulty constraining visual encoding depth and distribution in relation to task-objectives should be generally associated with greater RH activation, namely within the TPJ component of the VAN.

#### Nodes 1–2–3–4

In the course of performing task-directed actions, once task-relevant information has been perceptually identified via the proposed functioning of nodes 1, 2, and 3, the identified perceptual content should then be translated into verbal codes that can be efficiently integrated with, and used to update, task-directives in VWM (or the verbal cognition work-space). This section examines possible mechanisms associated with this process.

Language development studies show there is a transfer from right to LH processing of visual information that coincides with the learning of “name codes” (43). This also occurs in adults during the learning of novel visual items or concepts (44). Nevertheless, the extent and nature of hemispheric specialization for language remains a point of significant debate. Contributing to this is the fact that the broad concept of communication, which clearly involves non-verbal aspects, is often conflated with the more specific domain of linguistic operations. Moreover, we expect confusion has also stemmed from a failure to clearly delineate perceptual, motoric-articulatory, and semantic aspects of linguistic functions. Recent work by Hickok and Poeppel has helped to clarify such matters.

Through exhaustive integration of previous research and their own empirical studies, Hickok and Poeppel (45) suggested that linguistic operations can be divided into a bilateral ventral stream that manages sensory encoding and comprehension functions, and a left-lateralized dorsal system that translates linguistic signals into motor-articulatory codes that can be explicitly represented within frontal brain systems. This left-lateralized component is thought to be initiated via a temporal–parietal brain region critical for linking sensory content to verbal articulatory codes, and then via pre-motor and inferior frontal and insular regions that facilitate the integration of the phonetic-articulatory coded information into frontal systems, namely VWM. We conceive that this latter dorsal-stream aspect of linguistic operations comprises the primary operations of the fourth-node of our proposed TD-APS brain system. It is where identified task-relevant perceptual sensory content gets “verbally coded” (i.e., indexed via articulatory codes) and utilized to update task-directives in LH VWM (or the verbal cognition work-space). Moreover, we expect this operation is optimized with respect to task-objectives when verbal articulatory encoding is able to occur directly following initial “vision at a glance” operations as described above.

#### Additional considerations

Nodes 1 and 4 (VWM and verbal encoding) represent a left-lateralized aspect of the TD-APS brain system that serve its internal objective, while nodes 2 and 3 (predictive modeling and perceptual encoding) represent a right-lateralized aspect that serves its external objective. This internal/external dichotomy is considered an important feature of the proposed TD-APS brain system for the following reason. The primary goal of generating and updating plan sequences is fundamental to the goal of aligning visual processing operations with task-directives. Accordingly, if the internal goal is compromised, for any reason, the secondary external goal should not be well realized, resulting in greater explicit perceptual encoding of task-extraneous content, with an associated increased RH contribution during task-operations (i.e., visual sensory overflow). Indeed, because the primary external objective (i.e., task-directed visual sensory encoding) is considered the final convergent output function of the TD-APS system, any problem within this system should be reflected in this aspect.

Moreover, we believe this internal/external dichotomy may be linked to additional important EF network and neurochemical considerations. For example, although the default mode network (DMN) has been previously understood as a “resting” or “task-negative” system (46), recent studies indicate it may play a more finessed role in internally directed self-referential aspects of cognition, even during task-operations (47), and as such, may be involved with the internally oriented aspects of the proposed TD-APS system. Furthermore, the identified fronto-parietal EF-control network [for review see Ref. (5)] has been suggested to possibly mediate interactive dynamics between internally directed DMN and externally directed DAN and VAN brain functions (47, 48). Similarly, LH-internal/RH-external dichotomies have been previously described in relation to dopamine and norepinephrine functions (49, 50), and as a basic feature of human brain functioning (51).

In summary, we expect task-directed brain function depends on a specialized TD-APS brain system that uniquely facilitates task-directed actions via: (1) VWM (or verbal cognition work-space) to sequence, maintain, update, and initiate plan directives (with possible support from SWM to model integrated plan steps), (2) SWM (or perceptual cognition work-space) to generate predictive sensory models to help bias down-stream processing toward task-objectives, (3) perceptual identification of task-relevant items, and (4) translation of those items into verbal codes that can be efficiently integrated with, and used to update, plans in VWM. This system has the primary internal goal of organizing and updating task-plans and contingencies (directly mediated by nodes 1 and 4, with support from node 2), and the primary external goal of aligning visual encoding operations with task-objectives (directly mediated by nodes 2 and 3, with support from node 1). Moreover, we expect its induction and performance involves active regulation of automatic sensory responsivity, and that the DMN may play a key role in internal oriented processing, while the dorsal and ventral attention networks may help facilitate externally oriented processing, with the FPN possible helping to mediate and integrate between domains. Lastly, any degradation of this system’s operational fidelity is expected to manifest increased explicit processing of task-extraneous visual content, with an associated increased RH contribution during task-operations, namely in the RH TPJ aspect of the VAN. We will now attempt to re-frame ADHD pathology within the context of this proposed system.

### TD-APS distributed effects and ADHD

Before beginning this section, we feel it is important to highlight the relationship between our above presented model and Barkley’s seminal work on ADHD (see Box 1).

Box 1 **Revisiting Barkley.**

With a few clarifications, our TD-APS approach can be viewed as a visual sensory extension of Barkley’s original model of ADHD (41). Barkley essentially operationalized ADHD as an impaired ability to volitionally pursue future-oriented goal-directed actions, and characterized a set of integrated brain functions that underlie that ability (i.e., working memory, affect regulation, internalization of speech, and reconstitution), which he called the “motor control/fluency/syntax” (MCFS) system. Barkley also emphasized behavioral inhibition (BI) as being particularly important to this system’s operations. However, this point needs clarification in order to portray how our models interrelate.

Barkley’s emphasis on BI was influenced by Jacob Bronowski’s work on the evolution of language. Here, the capacity to inhibit automatic stimulus-response circuitry was viewed as a necessary pre-cursor to create a neuro-computational space within which volitional higher-order linguistic and associated cognitive abilities could evolve. Barkley anchors his model in this perspective by suggesting that the general suppression of pre-potent responsivity is necessary to set the stage for EF operations that serve goal-directed actions. However, in his model, he also points out that cognitive and behavioral responses to task-extraneous stimuli must be actively suppressed during task-operations, and indicated that this is accomplished via the emergent capacities of the MCFS system. This task-specific form of BI cannot logically be considered primary to the same system from which it emerges. Thus, in our reading of Barkley’s model, we take his assertions about the primacy of BI to refer specifically to the general form (i.e., general non-task-specific BI), and suggest that this point has been frequently confused in research aiming to vet the merits of Barkley’s work.

Viewed in this way, general BI of pre-potent responsivity is consistent with our notion of adaptive processing states, whereby specialized brain function modes must be maintained in service to different genres of cognitive and behavioral challenges – in this case, a task-oriented state that is mutually exclusive to pre-potent responsivity. We have described this same dynamic in relation to the TD-APS state, but clarify that the suppression of pre-potent *sensory* responsivity is considered to be an intrinsic feature of the TD-APS itself. Further confusing this dynamic is that among the described emergent MCFS operations, task-specific BI plays a unique role. It operates at the computational juncture where the internal machinations of Barkley’s proposed MCFS system generate external behavioral outputs. In other words, in Barkley’s model the task-adaptive constraining of motoric output is the primary *external* systems goal. Accordingly, any deficit to the MCFS system, no matter the cause, should manifest poor task-specific BI with an associated overflow of motoric activations. Considered in this way, and consistent with our work, poor task-specific BI is a *convergent deficit effect* of the MCFS system, while general BI of pre-potent responsivity reflects a fundamental state condition linked to the induction and application of the emergent MCFS brain system.

With these clarifications, as noted, our model is merely a visual–sensory version of Barkley’s work. The MCFS system was conceived in relation to the external goal of aligning motoric output with task-objectives, and its failure, no matter the cause, results in “motoric overflow.” Our conception of the TD-APS brain system was developed with respect to the external goal of aligning visual sensory encoding with task-objectives, and its failure, no matter the cause, is expected to result in “visual sensory overflow.” A comprehensive treatment of this general topic should 1 day integrate both motoric and visual domains.

#### The TD-APS brain system and ADHD brain function pathology

The most consistent aspect of identified ADHD brain function pathology is arguably its lack of consistency and specificity. Abnormal brain structure and function spans all four cerebral lobes (52, 53), while cognitive and clinical symptoms are surprisingly non-specific (52), and although abnormal response inhibition has been considered a primary deficit, multiple impairments are now identified that counter a “singular executive dysfunction” model (54, 55). In fact, deficits related to each of the above described TD-APS nodes are now identified, for instance: node (1) impaired VWM (56), node (2) impaired SWM (56), nodes (1–2–3) impaired selective attention (57–60), and behavioral inhibition (42, 61–63), which may reflect weakened RH frontal–parietal circuitry critical for task-control over visual encoding, node (3) impaired perceptual encoding (64–66), and node (4) slow naming speeds (54, 67–73). Moreover, abnormal EF-level network functioning has also been implicated in ADHD, involving the: DMN, FPN, and dorsal and ventral attention networks (5). As noted above, we expect these networks play key roles within the proposed TD-APS brain system. Lastly, it is important to note that these identified impairments do not typically rise to a level of severity within individuals that warrants an alternative primary diagnosis (i.e., other than ADHD), or that could be used as a basis for identifying ADHD. Instead, they are detectable at a group level and show considerable variability both within and between individuals (54, 55), consistent with the notion of “poor tuning effects.”

Even among psychiatrically healthy individuals the TD-APS brain system’s capacity is conceived to vary with moment-to-moment fluctuations in top-down regulation of its state integrity and constituent operations. Moreover, TD-APS operational variability is expected to reflect normally occurring subclinical weaknesses among constituent operations that, through applied effort, must be regularly and actively compensated for in order to maintain a robust task-orientation. Otherwise, decrements in the capacity of any constituent operations and/or the ability to compensate for such weaknesses is expected to increase task-associated performance variability, with an increased expression of intermittent TD-APS system lapses (i.e., going “off-task”). In short, impairment to this system is conceived to be a dynamic, functional, and emergent phenomenon that builds over time, and is impacted by moment-to-moment variability in top-down control.

In this vein, and consistent with the above highlighted variable ADHD pathology, we conceive that ADHD TD-APS impairment is likely to be a moving target that reflects variable mixtures of: (1) more severe “initial weaknesses” among TD-APS constituent operations (modular effects), (2) a reduced capacity to compensate for such weaknesses (functional-emergent effects), and/or (3) a lack of adaptive use of the TD-APS for psychosocial reasons (use effects) (Table 1). With any mixture of these, TD-APS brain system impairment is expected to occur more frequently, and include: (a) variable deficits of constituent operations, (b) more severe cascading secondary deficit effects, and (c) more frequent system-wide lapses, which together undermine TD-APS operational fidelity and manifest ADHD symptoms. Moreover, these deficit outcomes are expected to be revealed as performance “tendencies” that occur during task-operations. In short, under our conceptual approach, ADHD is expected to be associated with variable impairments, stemming from variable sources, which get variably expressed during task-operations, and we think this type of “deficit circumstance” may be an inherent feature of more dimensionally or quantitatively defined disorders (i.e., that are reflective of “poor tuning” versus “flat tire” effects).

##### Contending with ADHD clinical heterogeneity

No matter how the proposed TD-APS brain system gets disrupted, we expect poor system functioning to manifest around its primary internal and external objectives. Accordingly, two primary convergent deficit effects should be ubiquitously express in ADHD. First, there should be problems with task planning and EF-control operations linked to WM (i.e., impaired primary internal objective), and next, there should be an increased explicit perceptual encoding of task-extraneous content during task-operations or “visual sensory overflow” (impaired primary external objective). Moreover, each of these TD-APS convergent deficit effects should be generally associated with greater performance variability during task-operations, which can be considered an additional third and general form of TD-APS convergent deficit.

If such convergent deficit effects are substantiated, they may prove useful in helping to further elucidate more specific underlying causal factors (i.e., core deficits) among ADHD individuals. This could be done, for instance, by testing the association between convergent deficit effects and various constituent operations comprising the TD-APS, including EF network functions. Moreover, even without knowing exact causes, elucidating whether an individual’s impairments were primarily linked to modular, functional-emergent, or APS levels of brain functioning might help generate more deficit-centric treatments. Finally, novel treatments targeting convergent deficit effects may prove generally beneficial, for example, by increasing the TD-APS system’s overall capacity to compensate for transitory weaknesses and thereby minimize the scope and intensity of cascading secondary effects (i.e., deficit flows).

Although still inconclusive, multiple factors support the existence of ADHD convergent deficit effects. Several studies have now identified WM to be a vital hub in ADHD cognitive pathology (60, 61, 74, 75). It is also now well established that performance variability is a key feature of ADHD (55), with some suggesting it may reflect abnormal interactive dynamics of EF-level network functions (task-positive and task-negative networks) [for review see Ref. (76)]. Lastly, although it has been less well popularized, multiple studies, and even comprehensive reviews of imaging literature, have indicated that atypically increased reliance on RH contribution is a stable feature of ADHD, and as belabored above, we believe this is consistent with atypically increased explicit perceptual encoding of task-extraneous content during task-operations (i.e., visual sensory overflow).

We began examining the possibility of RH biased sensory processing in ADHD several years prior under the pretense that task-directed visual operations represented a special case of visual processing that is predominantly oriented toward making quick categorical discriminations, and that whenever this was compromised, no matter the cause, there should be an associated increased exposure to task-extraneous details (i.e., that are not required for making categorical judgments). Moreover, we reasoned that whatever internal brain functions supported task related cognition, task-directed visual encoding was an initial juncture at which those internal operations manifested their computational output, and as such, the relative fidelity of task-directed visual encoding operations might index the relative health of associated underlying brain functions. Under this view, we focused our ADHD research efforts on early-stage task-directed sensory encoding.

### The literature

To date, we have performed seven studies that directly examined lateralized contribution to task processing in ADHD, all of which produced strong evidence of RH biased sensory encoding (Table 2). We first identified this phenotype using lateralized signal-detection tasks. These produced several findings showing a clear bias toward RH sensory encoding, associated linguistic impairments, and abnormal interhemispheric interaction (77, 78), and further specified this pattern was partly mediated by top-down attentional resources (78), could result in advantages for RH specialized operations (78), and is evident across a large variety of task-operations (79). We then used fMRI and EEG to try to identify associated biomarkers and further characterize these effects. Here, we observed: ADHD non-verbal biased processing was evident during sub-executive operations (80), showed a unique developmental course among families heavily loaded for non-persistent ADHD (81), and stronger expression with increased ADHD family loading (81). Moreover, we found a robust and literature-consistent (82, 83) biomarker. ADHD subjects exhibited highly significant rightward beta (16–21 Hz) EEG asymmetry in inferior parietal brain regions during the Conner’s Continuous Performance Task (CPT) (84), which we have since replicated with the CPT and an additional SWM task (under review).

**Table 2:** **Summary of our previous studies of RH biased processing in ADHD**.

Title (year)	Sample	Method	ADHD results		Reference
Impaired linguistic processing and atypical brain laterality in adults with ADHD (2005)	21 ADHD, 22 control (adults)	Lateralized lexical decisions	–	Reduced sensitivity to word phonology;	Hale et al. (77)
			–	Increased sensitivity to word frequency;	
			–	Word impairment attributable to increased reliance on RH processing strategy	
Atypical brain laterality in adults with ADHD during dichotic listening for emotional intonation and words (2006)	19 ADHD, 22 control (adults)	Dichotic listening emotions and words	–	Reduced right ear (LH) task dominance;	Hale et al. (78)
			–	Better at detecting emotions;	
			–	Worse at detecting words;	
			–	Atypical responses only evident while attending to both ears (i.e., divided attention);	
			–	Less vulnerable to distractors targeting LH	
Atypical brain activation during simple and complex levels of processing in adults with ADHD (2007)	10 ADHD, 10 control (adults)	fMRI with forward and backward digit span tasks	–	Increased RH frontal and parietal activation during forward digit span;	Hale et al. (80)
			–	During backward task showed distributed effects implicating abnormal linguistic encoding and mental manipulation of stimuli	
Rethinking a right hemisphere deficit in ADHD (2008)	79 ADHD (children)	Assessed relationship between behavioral laterality and battery of cognitive task	–	Behavioral laterality taxing RH specialized processing with a requirement for interhemispheric transfer showed robust associations to multiple tasks;	Hale et al. (79)
			–	Behavioral laterality taxing LH specialized processing showed minimal associations	
Atypical alpha asymmetry in adults with ADHD (2009)	29 ADHD, 62 control (adults)	EEG alpha asymmetry at baseline and during CPT	–	Rightward alpha asymmetry in frontal and parietal brain regions;	Hale et al. (85)
			–	These were associated with ADHD symptoms (frontal-inattentive; parietal-hyperactive)	
ADHD familial loading and abnormal alpha asymmetry in children with ADHD (2010)	218 ADHD (children)	EEG alpha asymmetry at baseline and during CPT in ADHD children with and without ADHD parents	–	Greater ADHD family loading was associated with increased rightward alpha asymmetry in frontal regions, but decreased in posterior regions;	Hale et al. (81)
			–	ADHD children with ADHD remitted parent show unique age effect with increasing rightward parietal alpha asymmetry with age	
Atypical EEG beta asymmetry in adults with ADHD (2010)	35 ADHD, 104 control (adults)	EEG beta asymmetry during rest and CPT	–	Robust and highly significant rightward beta asymmetry in inferior parietal regions (P8–P7) during CPT, which showed abnormal association to other brain regions	Hale et al. (84)

*CPT, Conner’s continuous performance test; P8–P7 denotes the EEG anatomical where asymmetry indices were calculated*.

Several other research groups, using multiple methodological approaches, have also reported a general pattern of reduced LH and increased RH contribution in ADHD. Malone et al. (86) reported that ADHD individuals failed to demonstrate a normal LH advantage (suggesting relatively greater RH contribution) for a lateralized naming task that normalized with Methylphenidate, while Campbell et al. (87) reported that methylphenidate selectively slowed responses only to the stimuli expected to produce a RH advantage. Both studies concluded there was a baseline over-reactivity of right fronto-striatal circuitry in ADHD. Furthermore, it is now well established that ADHD involves linguistic processing abnormalities (77–79), particularly slow naming speed (54, 67–73), and careful consideration of imaging findings also supports a general pattern of reduced LH and increased RH contribution.

Functional imaging studies at rest or during simple (i.e., sub-EF) challenges show a pattern of reduced LH (88–91), and/or increased RH activations (80, 85, 92–94), which may reflect a default increased weighting of perceptual versus verbally mediated processing. Consistent with this, recent diffusion tensor imaging studies have reported increased RH parietal (95) and frontal (96) fractional anisotropy in ADHD, while recent structural studies have reported a lack of normally occurring L > R asymmetry of prefrontal cortical convolution complexity (97), and increased RH visual cortex volumes (98). Moreover, Franzen et al. (99) has shown that DMN function in ADHD involves reduced connectivity between posterior cingulate cortex (a primary DMN hub) and the right inferior parietal lobule, which might reflect a default reduced internal control over RH parietal operations. Finally, identified abnormal corpus callosum structure [for review see Ref. (52)] and function (100), as well as deviant left–right EEG coherence (92, 101, 102) clearly implicate abnormal integration of verbal and perceptually mediated brain functions in ADHD.

A general pattern of reduced LH and increased RH contributions also seems evident during more complex tasks; however, this literature is more variable, showing diffuse effects mainly consistent with variable weakness across the proposed TD-APS brain system (42, 103–105). Nevertheless, several imaging studies have shown an atypical association between behavioral performance and right-sided brain structure and function in ADHD (58, 106–112), while ADHD studies directly examining activation asymmetries and/or that directly compare left–right differences have more consistently shown R > L patterns, mainly in posterior brain regions (81–85, 92, 93). Moreover, a general pattern of low-left/high-right brain function in ADHD has been previously acknowledged by Fassbender and Schweitzer (113) who suggested, based on a review of ADHD brain imaging literature, that ADHD involved an increased reliance on neuroanatomy associated with visual/spatial and motoric processing (rather than linguistic processing) during active cognition; and more recently, a large meta analysis of ADHD functional imaging studies reported hyper-activation of the strongly right-lateralized VAN, noting it may be related to increased distractibility in this population (53), and Helenius et al. (114) has also implicated abnormal VAN functioning in ADHD.

We expect the lack of clear laterality effects in ADHD during more complex task-operations reflects several factors, such as: (a) RH bias with increased processing of off-task sensory content reflects a general information processing *tendency* that requires time-extended challenges to capture, (b) this characteristic is mainly detectable with direct comparison of left and right contributions (i.e., using asymmetry indices or direct left–right comparison), which is done infrequently, and (c) this phenotype is linked to a specific type of task challenge that is variably present across studies (i.e., rule based discrimination of visual targets versus task-extraneous content). Moreover, it is important to note that greater RH contribution with greater processing of off-task stimuli might occur in tandem with reduced RH activation linked to disrupted top-down influence and impoverished encoding of task stimuli. For example, right-lateralized aspects of FPN and DAN might be impoverished, while VAN and related bottom-up systems that are sensitive to stimulus saliency, novelty, and relevance, might be simultaneously over-activated.

Finally, in addition to the above findings, several ADHD characteristics are conceptually consistent with abnormal regulation of perceptual versus verbally mediated brain functions. For instance, ADHD is associated with increased leftward motor preference (115, 116), which has been linked to greater RH contribution to language (117, 118). ADHD is more common among males (119), who tend to exhibit better visual–spatial and poorer linguistic abilities compared to females (120, 121), and lastly, suspected low-dopamine and dysregulated noradrenergic function in AD5/20/14HD (122) may also align with abnormal R > L contribution as these systems appear to exhibit some degree of left and RH specialization respectively (49).

Together, our own research and the above noted literature clearly suggest there is some form of abnormal regulation and/or integration of verbal and perceptually mediated brain functions in ADHD. We have suggested that variable weakness within the proposed TD-APS brain system results in greater explicit perceptual encoding of task-extraneous content during task-operations (visual sensory overflow), and that this is reflected in greater RH relative to LH contributions to sensory encoding during task challenges. In our work, we have utilized the Conner’s CPT and EEG asymmetry, to identify a highly significant expression of increased RH contribution in ADHD (84). With this and our other consistent findings, as well as the above noted literature, we are now reasonably confident that this aspect of ADHD represents a real phenomenon and is an important convergent feature of the disorder. In future studies, we will utilize this phenotype to further examine the merits of our proposed model and explore how such convergent deficit effects might be utilized to help characterize different sources of pathology within the proposed TD-APS brain system.

## Model Predictions and Future Studies

With our current conceptual framework, any disruption to the proposed TD-APS brain system is expected to result in an increased expression of ADHD characteristics, with an associated increased expression of TD-APS convergent deficit effects, which are: (a) internal convergent effect = WM impairments, (b) external convergent effect = “visual sensory overflow” marked by greater RH contribution to sensory encoding during task-operations, and (c) general convergent effect = increased performance variability.

As noted, each of these characteristics is established as a feature of ADHD. However, our model further predicts they should more generally be associated with any neurobehavioral circumstance that is associated with increased expression of ADHD symptoms and characteristics, and/or increased risk of ADHD (e.g., being male, carrying ADHD risk-genes, anxiety, mood, left-handedness, reading disability, sleep deprivation, etc.). Accordingly, to test the merits of our model future studies should examine whether proposed convergent deficit effects are ubiquitously present across multiple ADHD risk factors, and/or whether they are generally associated with a greater expression of ADHD symptoms and characteristics.

If such associations are established, the next step will be to try to characterize possible variable sources of TD-APS brain-symptom impairment among different ADHD risk factors. Earlier, it was suggested that consideration of comorbidity profiles, stability of deficits, and quantitative versus qualitative impairments, might help to provide clues about the level of brain-function pathology disrupting TD-APS brain system functioning. Now that the model’s theoretical framework and proposed convergent deficits have been introduced, two additional tactics can be discussed.

First, unique causal deficits to the TD-APS brain system may result in variant forms of convergent deficit effects that might help to distinguish them. For example, with regards to parietal EEG asymmetry this could be reflected in variations of EEG frequency, exact region of effect, and/or variable strengths across different task challenges. Next, convergent deficit effects might also show different patterns of association to constituent cognitive operations comprising the TD-APS brain system. For example, in a population with primary linguistic impairment, TD-APS convergent deficit effects should show robust associations to measures tapping the impaired linguistic ability.

Although a dizzying array of factors could, in theory, underlie disrupted TD-APS brain system functioning (as we have conceptualized it), we remain hopeful that a limited set of primary causes might account for the bulk of ADHD diagnoses. With a clearer grasp of such causal deficits, more deficit-centric remediation strategies might become possible, including direct cognitive training of primary impairments. However, and importantly, our theoretical framework also suggests remediation strategies that do not require elucidating fundamental deficits sources.

For example, with a better grasp of TD-APS brain system constituent elements and how they interact, we may be able to devise remediation strategies that generally improve its performance (i.e., give the system a tune-up). Such an approach might incrementally move from training constituent modular operations in order of their hierarchical dependence (if one serves as a building block for another), and then attempt to directly train emergent EF-level network functions that bind modular operations, and finally, exercise the induction and maintenance of the TD-APS itself. Such an approach might be augmented by TMS based stimulation methods.

Another approach may be to directly target convergent deficit effects. These proposed common impairments may be an important source of negative secondary cascading deficit effects within the TD-APS brain system. Imagine a complex multi-stepped task-operation where, with each iterative step, diminished WM function, “visual sensory overflow,” and performance variability adds “computational noise” to the system. Over time, negative distributed/cascading effects resulting from this outpace the system’s compensatory abilities and produce a complete breakdown of the system (i.e., complete off-task distraction). If this hypothetical scenario is accurate, remediation strategies targeting convergent, rather than core deficits might generally increase TD-APS brain system resistance to such negative cascading effects and thereby produce a degree of clinical benefit regardless of original deficit sources.

## In Closing

The model, we have presented is highly theoretical and far from complete. Abundant work remains to both vet its assertions and further delineate and characterize its proposed components. We expect it to change and evolve. The intention of this work was to offer one possible starting-point for the continued investigation and refinement of whole-brain integrative conceptualizations of ADHD and other quantitatively defined psychiatric illnesses.

## Conflict of Interest Statement

The author declares that the research was conducted in the absence of any commercial or financial relationships that could be construed as a potential conflict of interest.
